# *PncA*gene expression and prediction factors on pyrazinamide resistance in *Mycobacterium tuberculosis*

**DOI:** 10.1186/1753-6561-8-S4-P17

**Published:** 2014-10-01

**Authors:** Katherine Lozano Untiveros, Patricia Sheen

**Affiliations:** 1 Universidade Federal de Alagoas, Maceió, Brazil; 2 Universidad Peruana CayetanoHeredia, Lima, Peru

## Background

Mutations in the pyrazinamidase (PZAse) coding gene, *pncA*, have been considered as the main cause of pyrazinamide (PZA) resistance in *Mycobacterium tuberculosis*. However, recent studies suggest there is no single mechanism of resistance to PZA. The pyrazinoic acid (POA) efflux rate is the basis of the PZA susceptibility Wayne test, and its quantitative measurement has been found to be a highly sensitive and specific predictor of PZA resistance. Based on biological considerations, the POA efflux rate is directly determined by the PZAse activity, the level of *pncA *expression, and the efficiency of the POA efflux pump system. This study analyzes the individual and the adjusted contribution of PZAse activity, *pncA *expression and POA efflux rate on PZA resistance.

## Methods

Thirty *M. tuberculosis *strains with known microbiological PZA susceptibility or resistance were analyzed. For each strain, PZAse was recombinantly produced and its enzymatic activity measured. The level of *pncA *mRNA was estimated by quantitative real time PCR, and the POA efflux rate was determined. Mutations in the *pncA *promoter were detected by DNA sequencing. All factors were evaluated by multiple regression analysis to determine their adjusted effects on the level of PZA resistance.

## Results and conclusions

Low level of *pncA *expression associated to mutations in the *pncA *promoter region was observed in *pncA *wild-type resistant strains. POA efflux rate was the best predictor after adjusting for the other factors, followed by PZAse activity.

These results suggest that tests which rely on *pncA *mutations or PZAse activity are likely to be less predictive of real PZA resistance than tests which measure the rate of POA efflux. This should be further analyzed in light of the development of alternate assays to determine PZA resistance.
